# Validation of a Career Guidance Brochure for Student Nurses Using the Nominal Group Technique

**DOI:** 10.29024/aogh.4

**Published:** 2018-04-30

**Authors:** Miriam Chinkhata, Gayle Langley, Aceme Nyika

**Affiliations:** 1 Malawi College of Health Sciences. Private Bag 396 Chichiri Blantyre 3, MW; 2 Witwatersrand University, Department of Nursing Education, ZA; 3 Graduate Support Research and Innovation Department, University of Pretoria, ZA

## Abstract

**Background:**

Nursing is a career which, especially for male students, requires one making an informed choice prior to pursuing it. A mixed-method, qualitative study, investigating the professional socialization process of male student nurses in Malawi found that most students did not make an informed choice regarding their selected career. This resulted in some of them facing many challenges which compromised their academic performance, and in some cases, contributed to high attrition rates. An “unmet need” for career guidance was identified by the study participants. Further, the study participants made recommendations on the need to address the unmet need for career guidance. A brochure entitled “A career in nursing and midwifery in Malawi” was designed. This article reports on the validation of the brochure.

**Objective:**

The goal of the study was to validate the brochure utilising the Nominal Group Technique.

**Methods:**

The validation exercise was part of the mixed method study. Five steps of the Nominal Group Technique (NGT) were utilised, as expounded by Vigra-Atkins, aimed at evaluating the content contained in the brochure. Study participants in four nursing colleges (n = 37) participated by studying the draft brochure and identifying strong and weak areas for improvements. Responses were ranked based on group consensus.

**Findings:**

A total of five groups were involved in NGT discussions. Four of the five groups found the brochure relevant and motivating to prospective students. However, two groups suggested that pictures needed to be more gender sensitive.

**Conclusion:**

Through the NGT, the brochure was commended by the majority of the participants as being clear and relevant in terms of giving information necessary for a nursing career choice. Finally, the authors recommend the use of the NGT compared to other group-based research techniques, considering the benefits the NGT offers.

## Introduction

Although the nursing profession has historically been dominated by females, involvement of men in the nursing field dates back to the 11^th^ century [1]. Despite this, men were secluded from caring for the sick. The founder of modern nursing, Florence Nightingale, accepted only *lady nurses* [1,2]. This development contributed to stereotyping nursing as a feminine profession. The trend was reversed after the second World War when men were then formally accepted to be educated as nurses [2,3]. Nowadays, despite many men entering the profession worldwide [4], the dominance of females has continued into the 21st century as shown by various studies. For instance, it was reported that the proportion of male nurses was 5.6% in 2005 in Canada [5], 10% in 2003 in the United Kingdom [6] and 25% in 2000 in Jordan [7]. It means that gender has an impact on the nursing career choices. This is in line with a researcher who asserted that the gender of an individual can be a factor in career choice [8].

Parents, families, guardians, guidance teachers and career advisors in western countries play a significant role in career choices of their children by influencing them either positively or negatively [9,10]. Similarly, in South Africa, a study found out that family, self-learners and teachers were significant factors in determining children’s careers; and it was recommended that parents and teachers should not force but rather should support and guide their children in making the right career choice [11]. Further, the study researchers emphasised that schools should provide career guidance programmes because career guidance made a significant impact on an individual’s career choice. This calls for informed career choice and decision making on the part of the prospective professional. In nursing, for example, Cohen advocates for an accurate and up-to-date information about nursing to be communicated to prospective nursing students [12]. This would help them make an informed career choice; and that the task of choosing a career is seen to be part of the individual’s developmental process [8].

Benefits of making an informed career choice upon joining any profession cannot be over emphasised. Informed career choice prepares an individual psychologically for the profession in question. It gives an opportunity to clarify career/personal aspirations and/or misconceptions, if any, and prevents an unnecessary career change from taking place upon registering at a university [11]. This is what was called anticipatory sociology [13]. This is in-line with what another writer emphasised; that university students including nursing students should have enough knowledge and insight about their career choices as it is one of the most crucial decisions an individual makes in his/her lifetime [14].

Contrary to this, findings of a qualitative study undertaken by the first author of this article in Malawi in 2014, that focused on gender mainstreaming in nursing education, revealed that the majority of male student nurses did not make informed career choices prior to joining the nursing profession [15]. As a result, the male students faced a lot of challenges during and after training [15]. For instance, some participants experienced reduced motivation shortly after joining the nursing career while others opted for career change. Such challenges could hinder the growth of the nursing profession. One major contributing factor to the challenges faced by nurses, especially male nurses, is lack of career guidance to the prospective nursing students [16,17]. In an effort to address this factor, an information tool (in the form of a brochure) titled, “A career in nursing and midwifery in Malawi,” was developed. Development of the brochure was based on recommendations made by the study participants in the 2014 study in Malawi [15].

Malawi is one of the countries in the sub-Saharan countries located south of the equator. It is bordered to the north and northeast by the United Republic of Tanzania, to the east, south and southeast by the People’s Republic of Mozambique and to the west and northwest by the Republic of Zambia [18]. The country educates both male and female nurse midwives at professional and technical levels. Education of male nurse midwives in Malawi began in 1985 at the University of Malawi [19]. Gradually, enrolment of men into nursing within the country spread to all nursing and midwifery colleges. Due to other factors, such as, Malawi being a patriarchal culture, men still remain a minority in the country. The trend is similar regionally and globally, though culture may not be a factor in other countries. Use of career guidance tools in form of a brochure could help recruit and retain prospective nurse midwives.

This paper describes how the brochure was validated using the Nominal Group Technique (NGT) as expounded by Varga-Atkins [20]. The aim of the NGT used in this study was to evaluate the outlook and content of the brochure, thereby increasing ownership of the brochure by Malawian nurse midwives. It is hoped that principles of the NGT as described in the article could be of benefit to other researchers in future.

The Table 1 below captures some important issues covered in the brochure.

**Table 1:** Key issues covered in the brochure.

Introduction	Introduces the brochure and explains rationale for its development
Definitions	Nursing and midwifery are defined.
Activities undertaken by nurse midwives	Some activities undertaken by nurse midwives are explained and complemented with pictures taken in various sections at one of the central hospitals.
Career Opportunities	Career path and opportunities in the profession are outlined.
List of training institutions	A list of universities and colleges educating nurse midwives in the country is given with contact information.
Academic Requirements	Academic requirements for each cadre of nurse midwives are outlined.

## Description of the nominal group technique

There are a number of group–based research techniques which researchers use to determine peoples’ views on services and other specific issues [21,22]. The techniques include focus group discussions, brainstorming, the Delphi technique and the nominal group technique. The nominal group technique has more advantages than the rest [23]. For example, the technique ensures the group achieves consensus and action planning on a chosen topic [20,23].

Researcher bias is minimised because participants are directly involved during both data collection and analysis [21]. Each group participant is given an equal voice since the process encourages individual input in a non-judgemental environment [23]. In addition, feedback in terms of results can be given to group members immediately. In cases where senior and junior personnel belong to the same group, they all interact or operate at the same level without some being intimidated [20].

The technique was first developed by Delbecq, Van de Ven and Gustafson in 1975 to facilitate effective group decision-making in social psychological research [23]. Since then, the technique has been applied by many researchers among many fields as a research method or evaluation method [23]. However, a number of modifications from the original technique have been introduced by various researchers based on the specific issues being addressed [20].

Some of the studies that have utilized the nominal group technique have been conducted in radiography [24], education [25], social service and physiotherapy [23].

According to the technique, participants take part in a highly structured face-to-face meeting of a small number of people, such as 5–9 participants. Group sizes may vary, however, as some researchers choose to use larger group sizes [20,23,25].

The nominal group technique has five stages during the validation process, namely: introduction, silent generation of ideas, sharing of ideas, group discussions and voting/ranking stage [20,23,25]. The stages are expounded in the methodology section when explaining the validation exercise.

Despite having a number of advantages, there are a few guidelines to be followed when applying the technique. The guidelines are considered to be disadvantages [23]. They include the need to formulate clear and stimulating question/s which will help address the issues at hand. Participants should know the issue/s to be addressed so as to contribute effectively and represent their profession adequately. The facilitator should have explicit knowledge of the issue/s being addressed. Finally, the facilitator should have skills in implementing the group technique.

## Methodology

The development of the brochure and the validation exercise were part of the multiple methods employed by the first author in a mixed methods study undertaken during doctoral studies. Appropriate ethical principles were employed. These ensured safeguarding of study participants’ rights. Therefore, prior to conducting the study, approval was sought from the appropriate authorities as follows: The Postgraduate committee for Faculty of Health Sciences and Human Research Ethics Committee at the University of Witwatersrand in South Africa – certificate Number M130805; The Ministry of Health Ethics Committee in Malawi – certificate Number NHSR 1235.

Permission to access institutions in Malawi where data were collected was sourced from the institutional heads. Study participants were requested to submit written consent.

The sample comprised student nurses (n = 37) in four purposively selected nursing colleges from a population of N = 849. The colleges represented university, government, and faith-based nursing colleges. Inclusion criteria of study participants included; willingness to participate in a NGT for approximately 1½ to 2 hours, being above 18-years of age and written informed consent. The study participants met the researcher in an agreed designated place on the college premises. Each group was gender balanced. There were 6–8 students per group.

A pilot study on the NGT was carried out at one of the nursing colleges with eight student nurses. The number comprised four females and four men. The aim of the pilot study was, firstly, to assess the extent to which answers of the set questions could provide the information required to validate the brochure. Secondly, the pilot study was to help the facilitator familiarise herself with the process of undertaking a NGT with students prior to undertaking the main data collection exercise. The findings from the pilot study did not form part of the main study.

The exercise took place in one of the classrooms at each college. The venue provided a conducive environment, which enabled participants to sit in a semi-circle to encourage participation. Each participant was encouraged to contribute freely in a non-judgemental manner.

### Application of the nominal group technique in validating the brochure

During the validation exercise, participants assessed the general appearance/presentability and content of the brochure. Content refers to the information contained in the brochure. Finally, the study participants were also required to indicate areas for improvements. Validation of the brochure was based on the five stages as expounded by Varga-Atkins et al [20]. The following five stages were carried out during both the pilot and the main study.

#### a. Introduction and explanations

Students were welcomed and thanked for taking the initiative to avail themselves for the activity. An information sheet regarding the study was issued to each study participant. Expectations of what was required from the participants were explained regarding the nominal group exercise. Emphasis was made that each participant was at liberty to either participate or not without being discriminated against in any manner. The first stage lasted approximately 15 minutes.

#### b. Silent generation of ideas

Each participant was provided with a draft copy of the brochure and a sheet of paper which had three questions to be addressed after studying the brochure. Participants were asked to write down their responses without consulting or discussing their opinions with anybody. Each participant was requested to write a maximum of three responses to each question.

#### c. Sharing ideas

Participants were then invited to share the ideas or responses they had generated. The facilitator recorded each idea on a flip chart using the participant’s spoken words. The round robin process continued until all ideas were presented. At this stage, there was no debate about the items/ideas/responses given. Participants were encouraged to write down any new idea that might have arisen from what others shared. This process ensured that all participants had the opportunity to make an equal contribution and provided a written record of all ideas generated by the group. All responses that were common were merged. This stage took approximately 30 minutes.

#### d. Group discussion

Participants were given the opportunity to request an explanation or clarification on any idea presented. The facilitator’s task was to ensure that each person was allowed to contribute, and that discussion of all ideas was thorough, yet without spending too long on a single idea. It was important to ensure that the process was as neutral as possible and to avoid being judgmental and critical about or to anybody. The stage lasted approximately five minutes as there were no issues for clarification.

#### e. Voting and ranking

During this stage, the participants were requested to prioritize five items on their list of strengths, weaknesses and areas for improvement on the brochure. From the prioritized list, each participant was requested to independently identify their top five items on their list respectively by ranking them and awarding five points to the highest item and one point to the least (i.e., 5–1 points). The participants were advised to write down the prioritized list on paper and rank the items accordingly.

Each participant was requested to share their ranking and scoring. Scores were added up for each section. The sections were namely: strengths, weaknesses and areas for improvement in the brochure. The facilitator indicated the points against each response on a flip chart and the final order of ranked responses’ for the questions was calculated together. Thus, immediate results in response to the questions were known by each participant since the exercise was conducted in a transparent manner. Finally, participants were thanked for their contributions and for allocating their precious time towards the exercise. The process was replicated in all the four nursing colleges.

Upon undertaking the exercise in all the four nursing colleges, the researcher compared common responses in all the nursing colleges giving consensus on the evaluation exercise of the brochure as a career guidance material.

## Results

### Demographic profile

Of the total sample (n = 37), n = 18 (48.65%) were males and n = 19 (51.4%) were females. Age ranged from 18 to 40 Years.

Below are results of the NGT presented in table format. Upon analysing the brochure, the study participants gave their inputs on strengths and weaknesses of the brochure and areas requiring improvement. See below for the responses.

Tables 2 to 4 give common results on strong areas, weak areas of the brochure and areas which the participants proposed for improvement. The issues have been exclusively identified not based on total scores given by the participants but rather found to have been mentioned by each group.

The following gives combined results common in more than one NGT.

Table 2 shows that 4 of the 5 groups found the information in the brochure to be relevant and motivating to an individual aspiring for nursing and midwifery. Three groups of the five found that the information about nursing was clear, gender sensitive and that challenges of the profession were explained. Two groups found the design and layout of the brochure appealing.

**Table 2:** Common Strong Aspects of the Brochure.

Number of nursing colleges that provided similar inputs	Participants’ Input

3	Gives clear information about nursing
2	Design and layout good
4	Relevant and motivates an individual
3	Information is gender sensitive
3	Challenges are explained

Table 3 gives results of areas that were commonly found to be weak on the brochure by more than one group. These included: font size, gender insensitive pictures and challenges outlined.

**Table 3:** Common Areas of Weakness.

Number of nursing colleges that provided similar inputs	Participants’ Input

2	Font size very small
2	Picture not gender sensitive
3	Challenges not gender sensitive

Table 4 outlines issues that were commonly identified by the study participants in more than one group that needed to be improved on the brochure. Issues raised for improvements were considered when designing the final brochure. These included gender specific pictures and those issues which were perceived critical despite being mentioned by one group such as font size. However, proposals that required more space to be included on the final brochure and or college specific guidelines such as fee structure were not included.

**Table 4:** Common Areas for Improvement.

Number of nursing colleges that provided similar inputs	Participants’ Input

2	Add places where nurses can work for example rural health centres and non-governmental organisations
2	Pictures to be gender-sensitive

## Discussion

The aim of this study was to validate the brochure by evaluating the content contained in it as a career guidance material. Specifically, participants were required to assess the general appearance and content of the brochure and provide objective feedback based on strengths, weaknesses and areas requiring improvements. The NGT proved to be effective and user friendly in validating the brochure. The results revealed that the design was well conceived with a layout that managed to give clear and relevant information about nursing and midwifery. The information included challenges that may be faced upon joining nursing. Though some participants in three of the five groups indicated that the brochure was gender-sensitive, two of the five groups suggested that pictures needed to be improved by adding some more pictures of males thereby improving gender sensitivity.

The majority of the groups (four of the five), found the brochure to be relevant and motivating to prospective nurse midwives. This finding is critical considering that the brochure is aimed at complementing information on career guidance and counselling for nursing students in general and male nursing students in particular. By participating in the validation exercise and suggesting areas that needed improvement, the nursing students demonstrated interest in the brochure thereby endorsing it for use in career guidance and for use in counselling prospective nursing students especially male students.

Use of appropriate career guidance materials for students making career choices is critical and should not be underrated. Malawian culture is to a large extent patriarchal. The fact that nursing and midwifery is still regarded as a feminine profession means that prospective male nurse midwives may require comprehensive career guidance prior to choosing the female-dominated profession. Such guidance could help ensure adequate psychological preparation as well as the ability to adjust to a female-dominated social environment. The guidance information would enhance the ability of the prospective nursing and midwifery students to make informed decisions prior to joining the career [26], thereby preventing career change during course of training or after [11]. This is because informed career choice help in averting wrong career choice [27].

The brochure, validated in this study, considered some factors that could influence an individual’s career choice. For example, the brochure contained career guidance information relevant to prospective professionals. The brochure, if used as a means of mass media could be an important information resource during career guidance activities. This could influence school leavers in their choice of a career in nursing [28,29]. Thus, the brochure could be used as a career guidance material thereby helping some prospective male nurse midwives make an informed career choice in the female dominated profession.

### Limitations of the study

Firstly, nurse midwifery students were utilized as proxy in validating the brochure. It would have been ideal to obtain views of secondary school graduates because they are prospective college students. Secondly, graduates may, indeed, not be familiar with what constitutes nursing and midwifery. Thirdly, the results cannot be generalized owing to limitations associated with the NGT. However, researchers may utilize the principles of the NGT in validating other tools.

## Conclusion

Using the nominal group technique, the validation process has revealed that the content and appearance of the brochure are satisfactory. The brochure is appropriate, acceptable and useful for prospective nurse midwifery students in Malawi. On the other hand, it was recommended that the brochure be enriched with pictures depicting male nurses at work so as to be gender sensitive.

Finally, the authors recommend use of the NGT, compared to other group-based research techniques, considering the benefits the NGT offers.
